# The development and validation of a novel LC-MS/MS method for the simultaneous quantification of Molnupiravir and its metabolite ß-d-N4-hydroxycytidine in human plasma and saliva

**DOI:** 10.1016/j.jpba.2021.114356

**Published:** 2021-09-02

**Authors:** Alieu Amara, Sujan Dilly Penchala, Laura Else, Colin Hale, Richard FitzGerald, Lauren Walker, Rebecca Lyons, Tom Fletcher, Saye Khoo

**Affiliations:** 1 Department of Molecular and Clinical Pharmacology, University of Liverpool, Liverpool, L69 7SX, UK; 2 The Royal Liverpool & Broadgreen University Hospitals NHS Trust, Prescot Road, Liverpool, L7 8XP, UK; 3 Liverpool School of Tropical Medicine, Pembroke Place, Liverpool, L3 5QA, UK

**Keywords:** Antiviral therapy, Coronavirus, Molnupiravir, N4-hydroxycytidine, LC-MS/MS, Plasma, Saliva

## Abstract

In light of the recent global pandemic, Molnupiravir (MPV) or EIDD-2801, developed for the treatment of patients with uncomplicated influenza, is now being trialled for the treatment of infections caused by highly pathogenic coronaviruses, including COVID-19.

A sensitive LC-MS/MS method was developed and validated for the simultaneous quantification of MPV and its metabolite ß-d-N4-hydroxycytidine (NHC) in human plasma and saliva. The analytes were extracted from the matrices by protein precipitation using acetonitrile. This was followed by drying and subsequently injecting the reconstituted solutions onto the column. Chromatographic separation was achieved using a polar Atlantis C_18_ column with gradient elution of 1mM Ammonium acetate in water (pH4.3) and 1mM Ammonium acetate in acetonitrile.

Analyte detection was conducted in negative ionisation mode using SRM. Analysis was performed using stable isotopically labelled (SIL) internal standards (IS). The m/z transitions were: MPV (328.1→126.0), NHC (258.0→125.9) and MPV-SIL (331.0→129.0), NHC-SIL (260.9→128.9). Validation was over a linear range of 2.5-5000 ng/ml for both plasma and saliva. Across four different concentrations, precision and accuracy (intra- and inter-day) were <15%; and recovery of both analytes from plasma and saliva was between 95-100% and 65-86% respectively. Clinical pharmacokinetic studies are underway utilising this method for determination of MPV and its metabolite in patients with COVID-19 infection.

## 1 Introduction

Emerging coronaviruses (CoVs) from animal reservoirs are causing severe and lethal disease in humans, currently with limited antivirals to treat these infections. This results in novel disease outbreaks, exemplified recently by the newly emerged SARS-CoV-2, the causative agent of COVID-19.

Nucleoside analogues which mimic naturally occurring nucleosides have been used to inhibit viral replication. Some of these compounds have been quite successful in the treatment of several viral infections. Yet others, including mutagenic nucleoside analogues, such as 5-fluorouracil and ribavirin, have failed to inhibit CoVs, possibly due to the proofreading activity of the viral 3’-5’ exoribonuclease (ExoN) [1].

There is a growing list of both novel and repurposed antivirals being tested for the treatment of COVID-19. Early in the pandemic, a number of antiretroviral drugs were investigated. Among these, protease inhibitors lopinavir/ritonavir and also darunavir/cobicistat have been trialled, but unsuccessfully, in patients with COVID-19 [2, 3].

Remdesivir, originally developed for the treatment of Hepatitis C and Ebola, has now been approved by the FDA for treatment of COVID-19 infection. Studies showed that the drug accelerated recovery among people hospitalized with COVID-19. However, the drug must be given intravenously (requiring hospitalisation), is difficult to manufacture, and is also expensive[4].

Other antivirals currently under investigation include nitazoxanide, originally used as an antiprotozoal agent that was later identified as a first-in-class broad-spectrum antiviral drug for the treatment of influenza [5] and PF-07304814, a novel protease inhibitor prodrug that targets the SARS-CoV-2 virus’s ability to assemble and replicate [6].

Molnupiravir (MPV), also known as EIDD-2801/MK-4482, is an orally bioavailable isopropylester prodrug of the ribonucleoside analogue β-d-N 4-Hydroxycytidine (NHC also known as EIDD-1931) which exhibits antiviral activity against several RNA viruses. Pharmacokinetic (PK) profiling indicated that MPV is orally bioavailable in ferrets and nonhuman primates [7], with its active metabolite NHC, demonstrating broad-spectrum antiviral activity against SARS-CoV-2, MERS-CoV, SARS-CoV, and related zoonotic group 2b or 2c bat-CoVs. It also showed increased potency against a CoV bearing resistance mutations to the nucleoside analogue inhibitor Remdesivir. Prophylactic and therapeutic administration of MPV, in mice models of infection with SARS-CoV or MERS-CoV, showed significantly improved pulmonary function and reduced virus titre and body weight loss [8].

Following oral administration of MPV, exposure to NHC was generally dose-proportional in all species. MPV is unstable in plasma and is rapidly converted to NHC, which in contrast, is much more stable in plasma (t_½_>6.5h). Once NHC is absorbed into animal plasma it is widely distributed in tissues where rapid conversion to the active NHC-5’-triphosphate (EIDD-2061) occurs. The primary mechanism of action of MPV is inhibition of viral RNA replication by incorporation of the NHC monophosphate metabolite into the viral RNA genome resulting in induction of viral error catastrophe [1, 9]. In addition, the active metabolite, NHC-5’-triphosphate, may act directly as a chain terminator to arrest replication.

A literature search for the analytical methods for these compounds shows limited results, with none clearly validated. Plasma levels of individual drugs have previously been quantified using liquid chromatography/tandem mass spectrometry (LC-MS/MS) [10], in animal models [11], but the validation details have not been fully published. Also, to our knowledge, there are no methods for quantifying saliva by LC-MS and furthermore, no simultaneous method for both analytes exists.

We present here a simple tandem mass spectrometric method for simultaneous quantification of MPV (EIDD-2801) and NHC (EIDD-1931) in plasma and saliva, using protein precipitation extraction, validated in accordance with EMA and FDA guidelines [12]. This method has been developed specifically for use in the Accelerating COVID-19 Drug Development – Phase I/II trial platform (AGILE) CST-2 trial, investigating EIDD-2801 and EIDD-1931 concentrations in subjects treated for COVID-19 [EudraCT 2020-001860-27].

## 2 Materials and Methods

### 2.1 Chemicals

MPV, NHC and their stable isotopically labelled (SIL) internal standards (^13^C^15^N_2_-EIDD-2801 and ^13^C^15^N_2_-N4-Hydroxycytidine respectively) were obtained from Alsachim SAS, Illkirch, France; methanol and acetonitrile (LC-MS grade) were obtained from Sigma–Aldrich. Deionized water was obtained from Duo Avidity Science water purification unit and further purified to 18.2 MΩ with the Purelab Ultra (Avidity Science Ltd., Long Crendon, Bucks., UK). Blank plasma was obtained from healthy drug-free volunteers from the National Blood Service (Speke, Liverpool, UK), with Ethics approval from the NHS Health Research Authority. Phosphate buffered saline (PBS 1x), obtained from Sigma–Aldrich, UK, was used as a surrogate matrix for saliva as others have previously done [13, 14].

### 2.2 Equipment

The chromatographic system was made up of an ExionLC AD Multiplate Sampler (temperature, 6°C) and an ExionLC AD pump (ABSciex Limited, Cheshire, United Kingdom). Analytes and SIL internal standards (IS) were eluted using a reverse-phase Atlantis dC_18_ column (3 μm:100 mm × 2.1 mm; Waters UK) housed in an ExionLC AD Column Oven at a temperature of 40°C. Detection and quantification using an ABSciex 4500 Triple Quadrupole (ABSciex Limited, Cheshire, UK) with a heated-electrospray ionization source. Tuning and data acquisition were carried out using Analyst 1.7 and processing/quantification using MultiQuant 3.0.3 (AB Sciex Limited).

### 2.3 Preparation of stock solutions, calibrators, QC samples and internal standards

Stock solutions were prepared from their respective reference standards in 100% methanol to obtain a final concentration of 1 mg/ml and stored at -80°C until use. Working solutions, containing varying concentrations of MPV and NHC, were prepared by diluting this stock solution in methanol to produce three concentrations (250, 6.25 and 0.25 μg/ml) which were then spiked into drug-free plasma or PBS. The internal standard (IS) working solutions for plasma (5 μg/ml of ^13^C^15^N_2_-EIDD-2801 and ^13^C^15^N_2_-N4-Hydroxycytidine) and saliva (2.5 ng/ml of ^13^C^15^N_2_-EIDD-2801 and ^13^C^15^N2-N4-Hydroxycytidine) were prepared in methanol.

Plasma and saliva working calibration standards were prepared from the three concentrations above by diluting in the appropriate volume of blank, drug-free matrix to produce a final concentration of 2.5, 5.0, 25, 62.5, 125, 750, 2500, 4000 and 5000 ng/ml.

Quality Control (QC) samples were prepared from the MPV and NHC primary stock. These consisted of High QC (4200 ng/ml), Medium QC (420 ng/ml), Low QC (6.3 ng/ml) and the lower limit of quantification (LLOQ; 2.5 ng/ml) for plasma and saliva.

Standards and quality control samples were prepared fresh on the day of analysis, to prevent analyte interconversion, using calibrated air-displacement pipettes.

### 2.4 Clinical Study set-up and sample collection

Following development and validation of our plasma and saliva assays, we analysed clinical samples from 18 subjects enrolled into a phase Ib clinical trial of molnupiravir. AGILE CST-2 trial [Clinicaltrials.gov - NCT04746183] is an open-label, Bayesian adaptive, seamless phase Ib/IIa trial to determining the optimal dose, activity and safety of molnupiravir for the treatment of COVID-19.

Subjects with PCR-confirmed SARS-CoV-2 infection who had mild-to-moderate disease and who were not hospitalised were enrolled within 5 days of developing symptoms and randomly allocated with a 2:1 ratio to either molnupiravir (administered twice daily (bd)) or standard of care. Molnupiravir dosing was increased in cycles (each with six subjects: four treated and two standard of care) at pre-specified dosing tiers of 300mg bd, 400mg bd, 600mg bd and 800mg bd orally; a dosing tier could be skipped if the Bayesian dose-toxicity model suggested this was safe. Details of the trial design are available in pre-print [15].

Blood was drawn at different time points after drug administration. Saliva and EDTA whole blood (2 ml) samples were collected from consented study subjects. Saliva was collected using Salivette™ tubes (Sarstedt Ltd, UK). In brief, the patient chews on the salivette swab for approximately 60 seconds after which the swab is returned to the Salivette and centrifuged to yield liquid saliva. Whole blood and saliva were collected on wet-ice until centrifugation (within 30 minutes of sample collection). In order to prevent the conversion of MPV to NHC, plasma and saliva supernatants were immediately (within 10 minutes of centrifugation) precipitated with acetonitrile (ratio = 3:1, acetonitrile:plasma v/v). Exactly 150 μL | 50 μL of plasma | saliva was added to pre-labelled 2 ml plastic cryovial tubes, containing 450 μL | 150 μL of acetonitrile and vortexed at ≥1500 rpm for 15 seconds. Sample extracts were immediately frozen at −80 °C until LC-MS analysis.

### 2.5 Sample preparation and extraction

Plasma and saliva working calibration standards and QC were prepared fresh on the day of analysis and were immediately precipitated with acetonitrile (at an equivalent 3:1 ratio as used to extract/stabilise the clinical samples). The standards, QCs and blank samples (150 μL plasma; 50 μL saliva) were aliquoted in duplicate into 5 mL glass test tubes, to each of which acetonitrile (450 μL plasma; 150 μL saliva) was added.

All extracted samples (blanks, standards, QCs and clinical samples) were mixed by vortexing and centrifuged (4000 rpm, 4°C, 5 minutes). Exactly 300 μL of the plasma supernatants (100 μL of saliva supernatants) were transferred to clean labelled 5 mL glass tubes followed by 20 μL of SIL-IS and these were dried under a nitrogen stream. They were subsequently reconstituted to a final volume of 100 μL in mobile phase and transferred to labelled autosampler vials that were tightly closed with crimp seals. The vials were loaded onto autosampler trays and 10 μL injected into the LC-MS system for analysis.

### 2.6 LC-MS Conditions

Chromatographic separation was achieved using a Waters C_18_ XBridge column (3.5 μm: 100 mm × 2.1 mm) and 1mM ammonium acetate solution adjusted to pH4.3 with acetic acid (mobile phase A) and 1mM ammonium acetate in acetonitrile (mobile phase B). A gradient elution method at a flow rate of 350 μL/min was used, with the temperature of the column set at T = 40 °C. Mobile phase gradient started with 98% mobile phase A, which was held for 1.2 minutes then increasing in organic content to 90% mobile phase B in 2.5 minutes. This was maintained up to 3.5 minutes followed by increasing the aqueous content back to 98% at 3.8 minutes and held at the initial conditions for reconditioning with a total run time of 6 minutes. The needle was washed with a mixture of methanol and deionised water (50:50; v/v) between injections.

The electrospray ionisation (ESI) mass spectrometer (MS) was operated in negative ion mode using selective reaction monitoring (SRM). The parameters for MS settings can be seen in Table 1. The m/z transitions for MPV (328.1→126.0) NHC (258.0→125.9) and labelled SILs MPV-SIL (331.0→129.0) NHC-SIL (260.9→128.9).

A weighted (1/x^2^) least square linear regression model was used to plot the ratio of peak area of analyte to peak area of IS against the nominal concentrations of the analytes in order to evaluate the calibration curves.

## 3 Validation methodology

### 1.1 Selectivity

Selectivity (using six different lots of blank human plasma) was determined by comparing the amount of background interference in relation to the assay lower limit of quantification (LLOQ). Area responses of interfering noise at the retention time of each analyte were accepted if the interference was less than 20% of the response of the LLOQ. Interferences/noise responses at the same retention time of internal standard were deemed acceptable if the % interference was less than 5% of the mean response of the internal standard areas in 6 LLOQ samples.

### 1.2 Accuracy and Precision

Three separate sets of accuracy and precision batches consisting of a calibration curve and LLOQ, LQC, MQC and HQC samples in replicates of six, were run. Inter-day (between-run) and intra-day (within-run) assessments were carried out. Assays were deemed acceptable if all the calibrants and QC samples were within ±15% of their nominal values (with the exception of the LLOQ for which ±20% was allowed).

### 1.3 Carryover

Duplicate injections of LLOQ (2.5 ng/ml) followed by blank samples and upper limit of quantification (ULQ; 5000 ng/ml) standards were used. Injections of ULQ were followed by 3 extracted blank plasma samples. The % carryover (in the extracted blank samples after ULQ) was calculated and expressed in relation to the assay LLOQ; the % carryover should not exceed 20% of the LLOQ concentration (EMA Bioanalytical method validation).

### 1.4 Dilution integrity

Analyte concentrations between 160-180% of the upper limit of quantification were spiked into various matrices and subsequently diluted 1:2 and 1:4 with blank matrices. The samples were then analysed, with concentrations from the standard curve (including the appropriate dilution factor) and compared against the expected nominal concentration.

### 1.5 Recovery and Matrix effects

Percentage recovery and matrix effects were determined quantitatively using the methods of Matuszewski *et al* [16]. Peak-areas of analytes at LQC/MQC/HQC concentrations from extracted plasma samples, were compared with the peak area of analytes spiked at an equivalent concentration in mobile phase to obtain % recovery (process efficiency). A comparison of the peak areas of the analytes spiked into blank plasma extracts with the peak areas of analytes in mobile phase at an equivalent concentration gave the % matrix effect. The IS normalised (analysis recovery) recovery was also calculated.

### 1.6 Stability

The stability of MPV and NHC in plasma (QC samples; 4 per level) was assessed at room temperature and on wet ice over 3 hours and following 1 freeze-thaw cycle spanning a period of 24 hours. Furthermore, assay autosampler stability was assessed by re-injecting an accepted precision and accuracy batch (6 QC per level) which had been left in the autosampler at 4°C for 48 hours. Reinjection reproducibility over 24 hours was also assessed.

### 1.7 Measurement of MPV and NHC in human plasma and saliva

Blood samples, for the pharmacokinetic analysis of MPV and NHC in the AGILE trial (EudraCT 2020-001860-27), were analysed using this method.

The data are being presented in a separate clinical manuscript.

## 4 Results and Discussion

**Figure 1:**
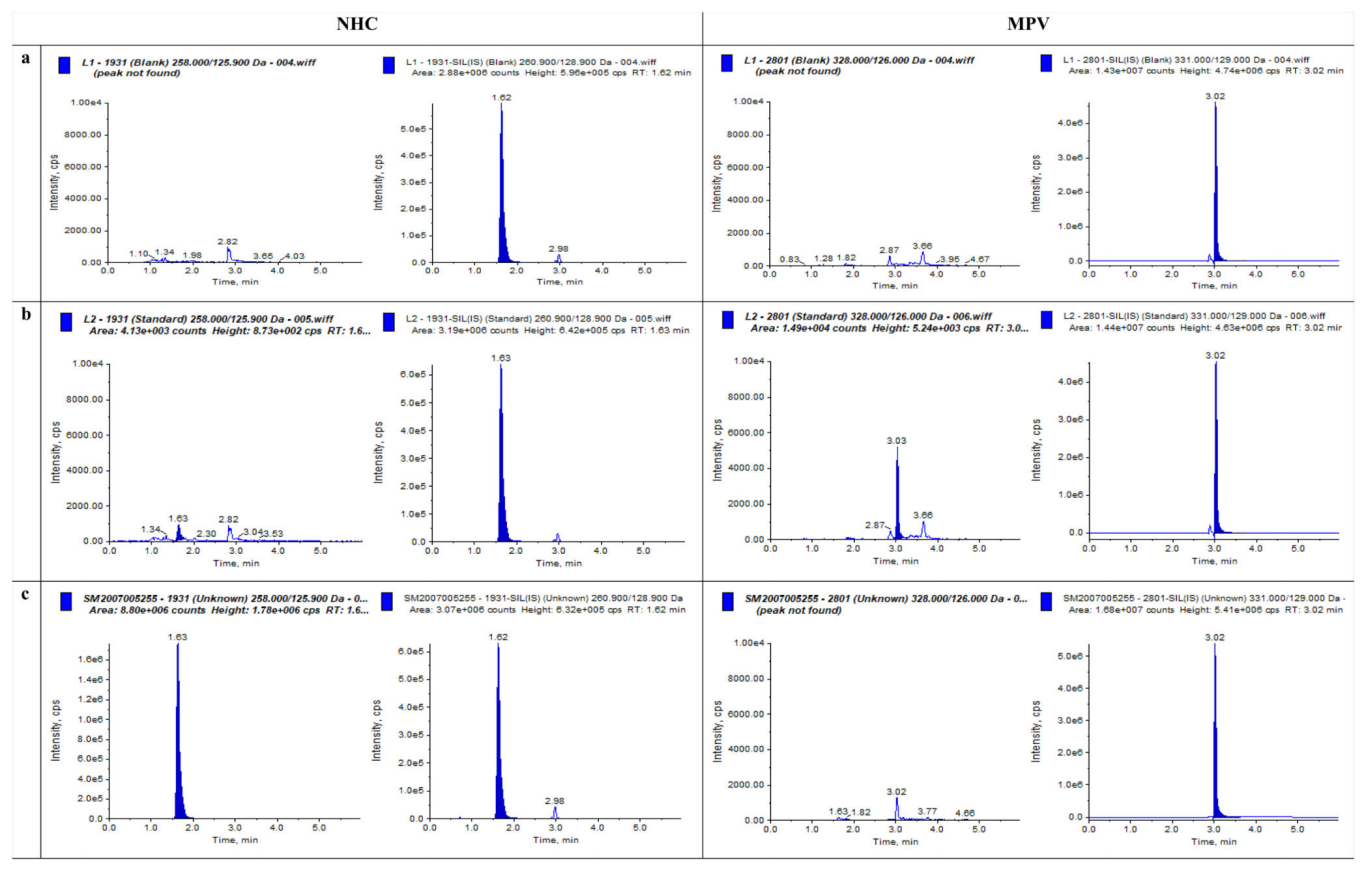
Chromatograms of NHC and MPV in **Plasma** in a) Blank extracted plasma sample; b) Spiked with *2.5 ng/ml* (LLOQ); c) Patient sample, *4245.7 ng/ml* **NHC**; **MPV** *<LLOQ;* all with internal standards

**Figure 2:**
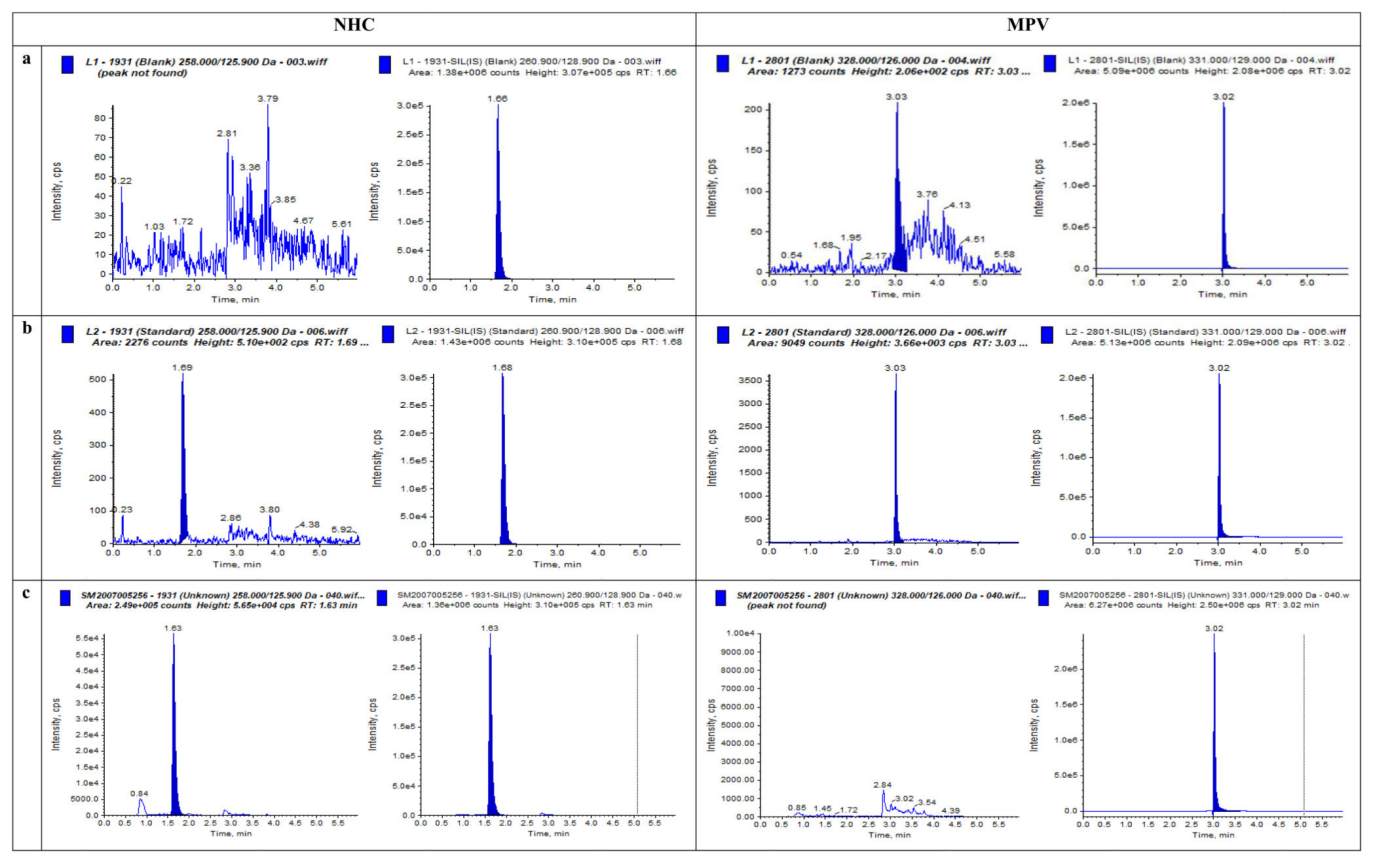
Chromatograms of NHC and MPV in **Saliva** in a) Chromatogram of Blank PBS sample; a) Spiked with *2.5 ng/ml* (LLOQ); c) Patient sample, *230.0 ng/ml* **NHC**; **MPV** *<LLOQ;* all with internal standards

MPV, NHC and their stable isotope labelled internal standards eluted from the HPLC column at 3.02 minutes and 1.62 minutes, respectively. A number of polar and non-polar C18 chromatographic columns were initially tried to resolve the analytes, including those by Fortis® and Ascentis®. However, the polar Atlantis® C_18_ column provided greater peak resolution and better peak shape. Representative chromatograms of extracted plasma and saliva samples (a zero sample with IS, LLOQ and clinical sample) are shown in Figures 1 & 2 **(a to c)**. Tuning and optimisation were attempted in both positive and negative ionisation modes but the negative mode was used as it provided greater signal intensity.

### 4.1 Method Validation

#### 4.1.1 Selectivity

The method was selective, revealing minimal background interference (<10% of the signal response at the LLOQ for MPV and NHC) in all six plasma batches tested.

#### 4.1.2 Accuracy and Precision

The accuracy and precision (both inter-day and intra-day) values at the assay LLOQ were within the designated ±20% and for all QC levels were within ±15% of the nominal values (Table 2) for both plasma and saliva extracts.

#### 4.1.3 Carryover

The mean % carryover (n=3) observed in the first extracted blank sample following injection of a ULQ sample (5000 ng/ml) was 54.8% of the assay LLOQ that, upon injection of the third blank plasma sample, reduced to 12.5% for MPV. Hence 2 blanks were incorporated into the assay sequence (between individual clinical samples, after the ULQ and each QC batch) in order to mitigate this. NHC had no carryover (equivalent to 5.9% of the assay LLOQ).

#### 4.1.4 Dilution integrity

Samples diluted by a factor of 2 and 4 times showed calculated concentrations within ± 15% of the nominal values for MPV and NHC.

#### 4.1.5 Recovery and Matrix effects

Process Efficiency (PE), Recovery Efficiency (RE), Matrix effects (ME) and Analysis Recovery were studied for both plasma and saliva matrices each at three different concentrations. Overall mean recovery is >90% in plasma with negligible matrix effects and >75% in saliva; the data can be seen in Table 3.

#### 1.7.1 Stability

The stability of MPV and NHC was assessed under a variety of conditions to facilitate the analysis of clinical samples and to eliminate the interaction between the two analytes. All stability experiments were conducted using plasma (for plasma samples). Stability data are presented in Table 4.

MPV, an ester pro-drug, is unstable in biological matrices where esterase enzymes are present, or where the pH exceeds 7.5 (at high pH, ester prodrugs hydrolyse). As a result, plasma/saliva samples require immediate stabilisation with acetonitrile in order to prevent conversion of MPV to its metabolite NHC (as described in section 2.4 (*Clinical Study set-up and sample collection*). Preliminary stability experiments, using plasma spiked with MPV and NHC in combination, confirmed this phenomenon. MPV was unstable at room temperature (>75% degradation) and even when kept on wet-ice (~20% degradation) for 3 hours. Initially, samples were processed on ice and samples were also treated with varying concentration of acid before analysis but these failed to make a difference (data not shown). Immediate addition of ACN was the final successful solution. Once acetonitrile was added to the plasma and saliva samples, the analytes were rendered stable.

Furthermore, extracted samples were also stable at room temperature and for up to 48 hours following injection within the LC-MS autosampler (4°C) with concentrations within ±15% of the respective nominal values.

#### 1.7.2 Clinical Application

Data and chromatograms from extracted plasma and saliva samples from patients receiving MPV are presented in Figures 1c & 2c. In the majority of the samples analysed (using our LLOQ of 2.5 ng/ml), the pro-drug MPV was unquantifiable in plasma and saliva or detected at low concentrations only at early timepoints (0·5 and 1-hour post-dose) in few samples. The metabolite NHC however was consistently quantifiable. In a set of 20 samples, the mean concentration of saliva and plasma NHC were 42.51 ±47.11 and 1040.37±734.23 ng/mL respectively. The mean saliva to plasma ratio was 0.12 ± 0.27. These data are purely descriptive; the complete pharmacokinetic data set is to be published in a separate clinical publication for the CST-2 AGILE trial.

## 2 Conclusion

We present here a sensitive, selective, accurate and robust LC–MS/MS method, developed and validated to quantify MPV and NHC in human plasma and saliva. This, to the best of our knowledge, is the first method quantifying MPV and NHC concentrations in saliva. This bespoke method, devised for the AGILE trial, can serve as the foundation method for other trials involving the same analytes.

Additionally, this assay will help provide greater clarity about MPV and NHC pharmacokinetics across different matrices under different treatment scenarios. The method is being used to study the pharmacokinetics of MPV and NHC in plasma and saliva as part of the AGILE clinical trial.

## Figures and Tables

**Table 1:** Source parameters for NHC and MPV

Parameter	MPV	MPV-SIL	NHC	NHC-SIL
**Mass Transitions (Da)**	328.0→ 126.0	331.0→ 129.0	258.0→ 125.9	260.9→ 128.9
**Collision Energy (V)**	-24	-24	-20	-20
**Spray Voltage (ISV)**	-4500	-4500	-4500	-4500
**Vaporizer Temperature (TEM°C)**	500	500	500	500
**Ion Source Gas 1 (GS1)**	50	50	50	50
**Ion Source Gas 2 (GS2)**	40	40	40	40
**Collision Gas (CAD)**	8	8	8	8
**Curtain Gas (CUR)**	30	30	30	30

**Table 2:** Precision and Accuracy for NHC and MPV in plasma and saliva in QC samples

	PLASMA					SALIVA			
NHC	LLOQ	LQC	MQC	HQC		LLOQ	LQC	MQC	HQC
**[Nominal QC] (ng/ml)**	**2.50**	**6.30**	**420.00**	**4200.00**		**2.50**	**6.30**	**420.00**	**4200.00**
**Mean value (ng/ml)**	2.34	5.80	444.75	4205.58		2.47	5.89	433.99	4499.80
**%CV: *inter-day***	*15.890*	*10.74*	*4.37*	*4.65*		*10.076*	*13.51*	*6.07*	*10.53*
**%Accuracy: *inter-day***	*-6.49*	*-7.89*	*5.89*	*0.13*		*-1.04*	*-6.57*	*3.332*	*7.138*
**%CV: *intra-day***	*6.88*	*9.84*	*3.20*	*3.05*		*7.94*	*7.92*	*2.96*	*0.99*
**%Accuracy: *intra-day***	*-4.53*	*-3.49*	*7.36*	*4.08*		*3.93*	*-14.10*	*4.97*	*7.29*
**MPV**	**LLOQ**	**LQC**	**MQ C**	**HQC**		**LLOQ**	**LQC**	**MQC**	**HQC**
**[Nominal QC] (ng/ml)**	**2.50**	**6.30**	**420.00**	**4200.00**		**2.50**	**6.30**	**420.00**	**4200.00**
**Mean value (ng/ml)**	2.61	6.22	467.89	4081.22		2.51	5.56	444.14	4321.29
**%CV: *inter-day***	*7.88*	*9.05*	*3.18*	*6.02*		*7.30*	*6.31*	*6.28*	*8.21*
**%Accuracy: *inter-day***	*4.31*	*-1.22*	*11.40*	*-2.83*		*0.56*	*-11.80*	*5.75*	*3.36*
**%CV: *intra-day***	*5.89*	*8.30*	*2.77*	*2.59*		*6.59*	*2.67*	*2.81*	*1.25*
**%Accuracy: *intra-day***	*-3.27*	*1.24*	*14.01*	*2.38*		*-4.87*	*-13.41*	*5.60*	*6.293*

Abbreviations: LLOQ lower limit of quantification; LQC, low quality control sample; MQC, medium quality control sample; HQC, high quality control sample; n=6 in 3 separate runs (inter); n=6 in 1 run (intra). Criteria for acceptable range of accuracy is ±15 % (±20 % at LLOQ).

**Table 3:** Recovery & Matrix of NHC and MPV in plasma and saliva

	PLASMA					SALIVA			
NHC	LQC	MQC	HQC	*%CV*		LQC	MQC	HQC	*%CV*
**%Process Efficiency**	*90.151*	*100.888*	*98.605*	*5.858*		*46.360*	*62.020*	*53.227*	*14.57*
**%Recovery**	*103.820*	*100.232*	*103.448*	*1.925*		*66.221*	*86.362*	*73.045*	*13.62*
**%Matrix Effect**	*86.834*	*100.654*	*95.319*	*7.393*		*70.007*	*71.814*	*72.869*	*2.022*
**%Analysis Recovery**	*98.394*	*90.917*	*104.542*	*6.966*		*72.446*	*109.383*	*84.809*	*21.155*
**MPV**	**LQC**	**MQC**	**HQC**	***%CV***		**LQC**	**MQC**	**HQC**	***%CV***
**%Process Efficiency**	*97.433*	*100.229*	*100.450*	*1.69*		*56.718*	*74.477*	*71.486*	*14.07*
**%Recovery**	*95.649*	*98.386*	*104.470*	*4.54*		*65.42*	*83.448*	*77.519*	*12.17*
**%Matrix Effect**	*101.865*	*101.874*	*96.152*	*3.302*		*86.691*	*89.250*	*92.217*	*3.094*
**%Analysis Recovery**	*79.216*	*81.612*	*94.339*	*9.557*		*65.330*	*94.104*	*82.326*	*17.951*

Abbreviations: LLOQ lower limit of quantification; LQC, low quality control sample; MQC, medium quality control sample; HQC, high quality control sample; n=6 in this experiment. %CV is the coefficient of variation.

**Table 4:** Stability of NHC and MPV (plasma only)

Stability		LQC	MQC	HQC
**Freeze thaw (1 cycle)** Plasma (-80°C)	**NHC**	0.11	8.35	3.55
	**MPV**	-8.91	-7. 90	-3.37
**Autosampler Stability (4 °C)** Processed (over 48 hours)	**NHC**	1.59	4.39	7.88
	**MPV**	7.14	0.04	1.69
**Reinjection Reproducibility (24 hrs)**	**NHC**	5.66	8.96	3.73
	**MPV**	-2.86	10.15	3.27

Abbreviations: LLOQ lower limit of quantification; LQC, low quality control sample; MQC, medium quality control sample; HQC, high quality control sample; n=6 in 3 separate runs. Values are expressed as % stability, compared against freshly prepared samples, with the exception of autosampler stability. Criteria for acceptable range of stability is ±15 %.
